# Antioxidation, Anti-Inflammation, and Regulation of *SRD5A* Gene Expression of *Oryza sativa* cv. Bue Bang 3 CMU Husk and Bran Extracts as Androgenetic Alopecia Molecular Treatment Substances

**DOI:** 10.3390/plants11030330

**Published:** 2022-01-26

**Authors:** Chiranan Khantham, Pichchapa Linsaenkart, Tanakarn Chaitep, Pensak Jantrawut, Chuda Chittasupho, Pornchai Rachtanapun, Kittisak Jantanasakulwong, Yuthana Phimolsiripol, Sarana Rose Sommano, Chanakan Prom-u-thai, Sansanee Jamjod, Chaiwat Arjin, Korawan Sringarm, Houda Berrada, Francisco J. Barba, Francisco David Carmona, Wutigri Nimlamool, Warintorn Ruksiriwanich

**Affiliations:** 1 Department of Pharmaceutical Sciences, Faculty of Pharmacy, Chiang Mai University, Chiang Mai 50200, Thailand; ckhantham@gmail.com (C.K.); pichchapa_li@cmu.ac.th (P.L.); tanakarn_c@cmu.ac.th (T.C.); pensak.j@cmu.ac.th (P.J.); chuda.c@cmu.ac.th (C.C.); 2 Cluster of Research and Development of Pharmaceutical and Natural Products Innovation for Human or Animal, Chiang Mai University, Chiang Mai 50200, Thailand; sarana.s@cmu.ac.th (S.R.S.); korawan.s@cmu.ac.th (K.S.); 3 Cluster of Agro Bio-Circular-Green Industry, Faculty of Agro-Industry, Chiang Mai University, Chiang Mai 50100, Thailand; pornchai.r@cmu.ac.th (P.R.); jantanasakulwong.k@gmail.com (K.J.); yuthana.p@cmu.ac.th (Y.P.); 4 Faculty of Agro-Industry, Chiang Mai University, Chiang Mai 50100, Thailand; 5 Lanna Rice Research Center, Chiang Mai University, Chiang Mai 50200, Thailand; chanakan.p@cmu.ac.th (C.P.-u.-t.); sansanee.j@cmu.ac.th (S.J.); 6 Department of Animal and Aquatic Sciences, Faculty of Agriculture, Chiang Mai University, Chiang Mai 50200, Thailand; chaiwat_arjin@cmu.ac.th; 7 Department of Preventive Medicine and Public Health, Food Science, Toxicology and Forensic Medicine, Faculty of Pharmacy, Universitat de València, 46100 València, Spain; houda.berrada@uv.es (H.B.); francisco.barba@uv.es (F.J.B.); 8 Departamento de Genética e Instituto de Biotecnología, Universidad de Granada, 18071 Granada, Spain; dcarmona@ugr.es; 9 Department of Pharmacology, Faculty of Medicine, Chiang Mai University, Chiang Mai 50200, Thailand; wutigri.nimlamool@cmu.ac.th

**Keywords:** androgenetic alopecia, anti-hair loss, anti-inflammation, bioactive compounds, *Oryza sativa*, 5α-reductase gene expression, rice bran, rice husk

## Abstract

Androgenetic alopecia (AGA), a hair loss disorder, is a genetic predisposition to sensitive androgens, inflammation, and oxidative stress. Unfortunately, current treatments with synthetic medicines contain a restricted mechanism along with side effects, whereas the bioactive constituents of plant extracts are multifunctional, with fewer side effects. The massive amounts of rice husk and bran are agricultural wastes that may cause pollution and environmental problems. Owing to these rationales, the local rice variety, Bue Bang 3 CMU (BB3CMU), which is grown in northern Thailand, was evaluated for the valuable utilization of rice by-products, husk (BB3CMU-H) and bran (BB3CMU-RB) extracts, for AGA treatment regarding antioxidant, anti-inflammatory, anti-androgenic activities, and the characterization of bioactive compounds. Our study verified that BB3CMU-H had the highest level of polyphenols, contributing to its greater antioxidant activity. Conversely, BB3CMU-RB was the predominant source of tocopherols, resulting in better anti-androgenic activities regarding the downregulation of steroid 5α-reductase genes (*SRD5A*). Notably, anti-inflammation via the attenuation of nitric oxide productions was observed in BB3CMU-H (0.06 ± 0.13 μM) and BB3CMU-RB (0.13 ± 0.01 μM), which were significantly comparable to diclofenac sodium salt (0.13 ± 0.19 μM). Therefore, the combination of BB3CMU-H and BB3CMU-RB could be utilized in cosmeceutical and pharmaceutical applications for AGA patients.

## 1. Introduction

Androgenetic alopecia (AGA), known as heritable hair loss, is a chronic progressive condition that can affect both men’s and women’s personalities [1]. AGA is baldness caused by the gradual miniaturization of genetically predisposed hair follicles, which are the target of androgen mediated by dihydrotestosterone (DHT). Testosterone is reduced to DHT, a potent androgen, by cytoplasmic 5α-reductase enzymes. DHT possesses a greater affinity towards androgen receptors compared to testosterone [2], leading to a gradual replacement of terminal hair with vellus hair and, finally, hair shedding [3]. In AGA-prone hair follicles, the accumulation of DHT promotes the miniaturization process [1]. DHT strongly binds an androgen receptor in the cytoplasm, forms dimerization, and then interacts with an androgen-responsive element depending on coregulators [2]. Moreover, there are several androgen-inducible negative mediators, including transforming growth factor -β (TGF-β), dickkopf-1, interleukin 6 (IL-6), and inducible nitric oxide synthase (iNOS) [2,4], which may affect biological activities in hair follicles [4].

In addition to genetic predisposition and androgens, microinflammation around hair follicles and oxidative stress is responsible for the pathogenesis of AGA [5]. The expression of inflammatory mediator and iNOS in dermal papilla cells was increased threefold after treatment with DHT [5]. This suggested that iNOS and nitric oxide (NO) are downstream effectors of androgen receptors in hair follicles [2]. Furthermore, the examination of biomarkers revealed that NO, nuclear factor-κB (NF-κB), tumor necrosis factor-α (TNF-α), TGF-β1, thioredoxin, and total oxidant status serum levels were significantly higher in serum obtained from AGA than in that from the control group [6].

Nitric oxide synthases (NOSs) convert L-arginine to L-citrulline and NO [7]. Remarkably, a basal level of NO has been identified to influence various physiological effects of skin such as wound-healing, vasodilation, hair growth, and melanogenesis [8,9]. However, NO can be a potent cellular toxin in the immune system [5]. The excessive accumulation of NO leads to oxidative damage, the prolongation of inflammation, and the propagation of immunological responses [5,6]. The expression of iNOS was found to be upregulated at the peripheral tissues of hair follicles, and subsequently generate NO, leading to an inflammatory response and the release of a multitude of cytokines [9]. Both chronic inflammation and oxidative stress contribute to vascular dysfunction, reducing the vasodilatory capacity, and creating a perturbation of blood flow [10]. Reductions in the blood flow that transfers oxygen and nutrients to hair follicles in balding areas is thought to cause AGA [11]. Therefore, herbal extracts and/or biological compounds that act as a scavenger of NO or inhibitors of NO production could be used to promote hair growth.

Oxidative stress is implicated in the pathogenesis of AGA relating to the premature senescence of dermal papilla cells, reduction in cell migration, and secretion of hair follicle inhibitory factors [12]. The interaction between oxidative stress and inflammation has been previously identified in skin appendages, notably hair follicles [3,12,13]. Excessive inflammatory responses contribute to an increase in free radical production and an enhancement of oxidative stress [10]. Prie et al., proved that the indicators of oxidative stress, including a decrease in total antioxidant activity and an increase in malondialdehyde levels, were found in plasma samples of patients with AGA [13]. The production and accumulation of reactive oxygen species (ROS) in hair follicles are induced by oxidative stress [3]. In addition, hair follicular keratinocytes respond to oxidative stress by producing NO and IL-1, which have been shown to impede hair growth processes [3].

The hair loss treatment was conducted by synthetic medicines for decades, for example, anti-androgen drugs (spironolactone, finasteride, and dutasteride), a potassium channel agonist (minoxidil), an anti-seborrheic agent (tretinoin), and an anti-inflammatory drug (corticosteroids) [14]. Nevertheless, their side effects have reduced their usage [15]. Recently, demand for alternative hair loss treatment using natural products has increased worldwide, which has led to enhanced interest in herbal medicines and natural drugs of natural origin [9].

Rice (*Oryza sativa* L.) is one of the most broadly consumed grains for over half of the world [16]. The composition of rice grains includes rice husk, rice bran, brown rice, polished or milled rice [17]. Rice husk and rice bran are the most abundant by-products, which generated approximately 20% and 10% of the total weight of whole rice in the milling process [16]. Since rice husk is a poor nutritive and inedible, its utilization and commercial interest are extremely low and it is mainly used in non-food and cosmetic applications [18]. Similarly, rice bran is commonly utilized as animal feed, fertilizer, and fuel production [16]. Even rice husk and rice bran are considered by-products; several studies reported that they contain a variety of minerals and bioactive compounds, including phenolic compounds (flavonoids and γ-oryzanol), polysaccharides, and tocopherols [19,20,21,22].

Interestingly, the non–glutinous local rice variety with premium nutritional quality grains, Bue Bang 3 CMU (BB3CMU), which is grown in northern Thailand, showed high mineral contents [20]. Moreover, their biological activities and applications in the development of cosmetics, nutraceuticals, and food supplements of BB3CMU rice have not yet been investigated. The valuable utilization of rice-by products would provide benefits for farmers and reduce the environmental pollution caused by their disposal [16]. Some studies confirmed the differences in the antioxidant potential of rice fractions; there is no previous study that identifies and compares rice husk and bran extracts in terms of their anti-inflammatory capacity and the modulation of 5-alpha reductase gene expression.

With regards to these, the transformation of agricultural residuals into highly valuable anti-hair loss products is essential, along with scientific support. This study aimed to determine the bioactive compounds of rice husk (BB3CMU-H) and rice bran (BB3CMU-RB) extracts and evaluate their effects on antioxidant activities, anti-inflammatory activity, and the regulation of gene expression of steroid 5-alpha reductase isoenzymes, verifying the potential application of BB3CMU-H and BB3CMU-RB in AGA treatment.

## 2. Results and Discussion

### 2.1. Extraction Yield and Bioactive Compound Estimation

*Oryza sativa* L. cv. Bue Bang 3 CMU (BB3CMU) is brown rice that has a straw-colored husk and white pericarp [20]. The extraction yield of BB3CMU-RB (8.49 ± 1.83%, *w/w*, dry material) was higher than that of BB3CMU-H (0.70 ± 0.55%, *w/w*, dry material). The physical appearances of both extracts were dark brown semisolid and greasy. BB3CMU is non-pigmented rice with a non-pericarp color [23].

As shown in Table 1, the bioactive compound estimation of BB3CMU-H was significantly higher than BB3CMU-RM regarding the contents of total phenolic compounds, total polysaccharides, and total proteins. The total phenolic content of BB3CMU-H (23.68 ± 0.56 mg GAE/g extract) was approximately two times higher than BB3CMU-RB (11.63 ± 0.40 mg GAE/g extract). This finding supports the previously reported data showing that the raw material of rice husk fractions had a greater phenolic content than other fractions, including bran, brown rice, and milled rice [24]. Nevertheless, total phenolic and total flavonoid contents varied between different genotypes of rice varieties and growth sites [17,24].

Higher amounts of polysaccharides and proteins were observed in BB3CMU-H than BB3CMU-RB. Husk and bran contain polysaccharides, proteins, and peptides, which are immune-enhancing compounds [25,26,27]. A previous study reported that rice husk fraction contained a high carbohydrate content and was used as dietary fiber [27]. β-glucan, which can be found in milled rice, rice bran, and rice husk, has been suggested to be a functional polysaccharide with immunomodulatory properties [26]. Rice husk polysaccharides consisting of arabinose, galactose, glucose, mannose, and xylose have been suggested to modulate immune responses [27]. Besides the phenolic compounds in rice, the contents of protein (albumin, globulin, glutelin, and prolamin) and polysaccharide fractions were confirmed to have antioxidant activities [19]. However, additional techniques, such as the enzymatic method and hot-water extraction, need to be implemented for the better extraction of polysaccharides and proteins from rice fractions [27,28]. BB3CMU-H contained higher contents of phenolic acids, flavonoids, polysaccharides, and proteins than BB3CMU-RB. A previous study proposed that the rice varieties, genetic variations, and growth sites could affect the distribution of bioactive compounds in each fraction [19,24].

### 2.2. Profile of Phenolic Compounds, Phytic Acid and Tocopherols of BB3CMU-H and BB3CMU-RB

Table 2 shows the contents of phenolic compounds, phytic acid, and tocopherols in BB3CMU-H and BB3CMU-RB. Phenolic compounds are a class of compounds found in many plant foods, including flavonoids and phenolic acids [19]. Polyphenols were most abundant in BB3CMU-H, whereas tocopherols were only detected in BB3CMU-RB. This was consistent with the bioactive compound estimation using the colorimetric analysis in the previous section. Another study also reported that total phenolic acids were found in rice fractions in the following, decreasing order: husk, bran, whole grains, and endosperm [19].

The three major phenolic compounds found in BB3CMU-H were *o*-coumaric acid (910.07 ± 1.03 μg/g extract), kaempferol (863.58 ± 2.03 μg/g extract), and catechin (295.38 ± 0.39 μg/g extract). On the other hand, the amounts of EGCG (174.62 ± 0.01 μg/g extract), chlorogenic acid (113.79 ± 0.01 μg/g extract), and naringin (113.18 ± 0.01 μg/g extract) were predominant in BB3CMU-RB. Additionally, the most abundant constituent in BB3CMU-H and BB3CMU-RB is phytic acid or myoinositol-1.2.3.4.5.6-hexakisphosphate. Phytic acid has been highlighted as a strong and important antioxidant in non-pigmented rice [19].

Phenolic acid compositions such as caffeic, chlorogenic, coumaric, and ferulic are hydroxycinnamic acid derivatives [19]. Isomers of *o*- and *p*-coumaric acids are predominantly present in rice husk due to their association with lignin in the cell wall [17,24]. A high amount of ferulic acid in cereals was found in kernel husk (75%) and grain endosperm (15%), and the rest was found in the aleurone layer [29]. There is controversy as to whether ferulic acid was mostly found in bran [24]. Gallic and *p*-hydroxybenzoic acids are hydroxybenzoic acid derivatives. Our result was consistent with previously reported results that gallic acid was mostly evident in the bran fraction of non-pigmented rice [19]. *p*-Hydroxybenzoic acid has been reported to be the major phenolic acid in rice husk and cereal grains, such as wheat, barley, and corn [29,30].

The distribution of catechins in rice fractions differed regarding the rice varieties [17]. Rice is abundant in the husk and bran fractions [19]. Catechin was particularly present in BB3CMU-H, whereas EGCG was detected in both extracts. Remarkably, flavonoids including kaempferol, naringin, quercetin, and rutin were preponderantly detected in BB3CMU-H. A higher amount of flavonoids was observed in pigmented rice varieties than in non-pigmented rice verities [19].

The structure of vitamin E homologs, including the hydroxy groups on the aromatic ring and the methyl groups on a chroman ring, can be attributed to their amphiphilic characteristics [31]. The differences in the number and position of methyl groups on the chromanol structure contributes to the isoforms of α-, β-, γ-, and δ-tocopherol [32]. It has been proposed that tocopherol contents are not associated with the color of rice [19]. BB3CMU-RB is a rich source of tocopherols, especially α-tocopherol. Similar findings of α-tocopherol as the major toco derivative were observed in Taiwanese rice varieties [33]. Vitamin E is mostly found in bran, followed by whole grain and endosperm [19].

Our results verified that BB3CMU-H provides the most phenolic compounds. This means that the rice husk can no longer be discarded as a valueless fraction of the rice grain. Besides, BB3CMU-RB is a rich source of gallic acid and tocopherols. These bioactive compounds might exert or modulate molecular mechanisms related to their beneficial effects on promotion of hair growth, including antioxidant, anti-inflammation, and/or anti-androgenic pathways.

### 2.3. Antioxidant Activities

The presence of oxidative stress in the dermal papilla cells taken from AGA was confirmed [12,13]. Dermal papilla cells from a balding scalp were more susceptible to environmental stress than those from a non-balding scalp [12]. Oxidative stress is a result of the imbalance between the production of ROS and antioxidants in cells. Reactive oxygen and nitrogen species are characterized by the presence of one or more unpaired electrons in their outer shells [10].

ROS in hair follicles is produced and accumulated in response to extrinsic factors such as smoke, inflammatory processes, androgenic stimuli, free metals, and drugs [1,34]. Even the levels of catalase and glutathione were elevated in the balding dermal papilla cells; neither total ROS nor levels of senescence were reduced [12]. This proved that the balding dermal papilla cells have an inadequate ability to cope with ROS. This could lead to a therapeutic role for antioxidants, counteracting oxidative stress related to hair loss.

The DPPH radical and ABTS radical cation are stable and universally used to study the free-radical-scavenging activity of various samples [35,36]. As an excess of free irons contributes to the induction and formation of free radicals in biological systems, metal chelating activity was also evaluated using the ferrous ion-chelating assay [37,38].

The radical-scavenging and metal-chelating activities of BB3CMU-H and BB3CMU-RB are presented in Table 3. BB3CMU-H had superior scavenging activities to BB3CMU-RB in DPPH and ABTS assays. On the other hand, BB3CMU-RB had a higher ferrous-ion-chelating activity than BB3CMU-H. A study reported that the raw materials of Thai rice bran and husk possessed a greater antioxidant activity compared to the raw materials of brown rice and milled rice [24]. The average DPPH-radical-scavenging activity of non-pigmented rice bran and husk was about 27 mg TE/g extract [19]. Furthermore, it has been suggested that rice husks contain various biologically active compounds that are beneficial to the antioxidant defense system to protect the rice seed from oxidative stress [18]. However, there is controversy regarding whether the ability to scavenge DPPH and ABTS radicals of the Thai rice bran fraction was higher than that of the Thai rice husk fraction [24].

The antioxidant capacity of fruits, vegetables, and beverages, analyzed by the ABTS method, was higher than using the DPPH assay. Since the ABTS assay is in an aqueous system, antioxidants with hydrophilic properties were better reflected by the ABTS assay than the DPPH assay [25]. The radical scavenging ability of extracts was dependent on the phenolic content in each fraction [24]. The chemical structure of phenolic acid comprises a carboxylic acid group and a phenolic ring. The latter can stabilize and delocalize unpaired electrons, contributing to an antioxidant property [19].

A previous study reported that the ferrous-chelating activity of non-pigmented rice bran was roughly 83 mg EDTAE/g extract and was higher than that of rice husk [19]. The metal-chelating capacity of α-tocopherol was reported to be similar to EDTA, BHA butylated hydroxyanisole (BHA), and butylated hydroxytoluene (BHT), but higher than the same concentration of trolox [37]. Even *p*-coumaric acid and caffeic acid stabilized the oxidized form of the metal ion; their ferrous-ion-chelating effects were lower than EDTA and α-tocopherol [39]. In addition, phosphorus compositions, including inositol polyphosphate, inorganic phosphorus, and cellular phosphorus in rice, are considered ferrous chelators. The phosphorus content in rice fraction is in the following, decreasing order: bran (21.56 mg/g), whole grain (4.16 mg/g), endosperm (1.67 mg/g), and husk (1.03 mg/g) [19]. This may explain the greater chelating ability of BB3CMU-RB compared to BB3CMU-H.

Bioactive compounds in rice extracts exhibited their antioxidant activities in a concentration-dependent manner [19]. Phenolic acids exhibited higher antioxidant activities than those of α-tocopherol, anthocyanins, and γ-oryzanol [19]. A recent study has been shown that phenolic compounds of rice bran extract have the potential to reduce oxidizing free radicals and suppress ROS formation in an oxidative stress environment through the inhibition of radical-producing enzymes and the enhancement of an antioxidant defense system [30]. In addition, hydroxide groups of polysaccharides serve as electron donors and stabilize free radicals, thereby terminating radical chain reactions [28].

Oxidative stress and inflammatory mediators are known to be vital factors for the development of the AGA, as well as chronic inflammation [1,8]. Oxidative stress ultimately leads to the expression of cetrtain genes or mediators, implicating the inflammatory pathways [40]. In non-pigmented rice varieties, the content of γ-oryzanol is highest in bran (3067.1 mg/kg), followed by whole grain (288.6 mg/kg) and endosperm (58.9 mg/kg), but is not detected in the husk fraction [19]. The antioxidant activities of BB3CMU-RB could be the cooperative effects of tocopherols and γ-oryzanol. Despite BB3CMU-H having an absence of tocopherols, it showed greater antioxidant activities. It was presumed that the high amounts of phytic acid, *o*-coumaric acid, kaempferol, and catechin might be the major antioxidant components of BB3CMU-H. Therefore, BB3CMU-H may help to reduce oxidative stress and contribute to the attenuation of the inflammatory cascade.

### 2.4. Anti-Inflammatory Activity

Inflammation is the result of an immune system overreacting and the overproduction of reactive oxygen species, reactive nitrogen species, and inflammatory mediators produced from macrophages and neutrophils, contributing to the aggravation of inflammation and excessive tissue damage [31,32]. The cascade of macrophages events during lipopolysaccharide (LPS)-induced inflammation involves the mitogen-activated protein kinase (MAPK) and NF-κB pathways [41,42]. Additionally, NF-κB is a crucial transcription factor for iNOS [43]. The activation of both pathways results in the production of different mediators, such as NO, prostaglandin E2 (PGE2), and pro-inflammatory cytokines IL-1β, IL-6, and TNF-α [30,44].

Cellular nitric oxide synthase (NOS) is divided into constituted NOS (cNOS), endothelial NOS (eNOS) and inducible NOS (iNOS). The latter is provoked by LPS, inflammatory triggers derived from gram-negative bacteria, and produces NO [45], whereas eNOS worked with vascular endothelial growth factors to promote angiogenesis and hair growth [9].

**Figure 1:**
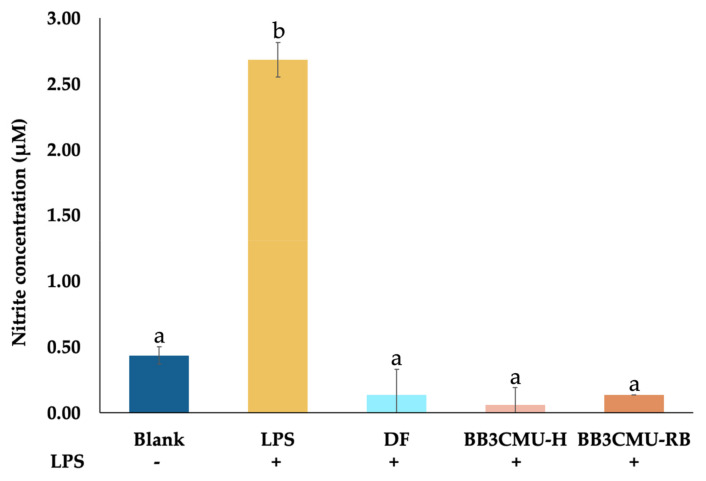
Effects of diclofenac sodium (DF), husk extract of *Oryza sativa* L. cv. Bue Bang 3 CMU (BB3CMU-H), bran extract of *Oryza sativa* L. cv. Bue Bang 3 CMU (BB3CMU-RB) at the equal concentration of 0.10 mg/mL on nitrite production in lipopolysaccharide (LPS)-stimulated RAW 264.7 murine macrophages for 24 h compared to vehicle-treated control without LPS (blank) and LPS-stimulated control (+LPS). Different letters indicate significant differences (*p* < 0.05): a and b indicate statistical significance in comparison to +LPS and DF, respectively.

In this study, a mouse macrophage cell line, RAW264.7, was treated with LPS to stimulate NO production. The quantification of NO was indirectly estimated from the metabolite nitrite. In Figure 1, the level of nitrite in the LPS-treatment group without extracts and diclofenac sodium (DF) were significantly elevated when compared with a vehicle-treated control (blank). Nevertheless, the inhibition of NO production was distinctly noticed in the groups pretreated with DF, BBCMU-H, and BB3CMU-RB. The nitrite production of cells treated with BB3CMU-H and BB3CMU-RB were reduced to 0.06 ± 0.13 μM and 0.13 ± 0.01 μM, respectively. DF reduced nitrite release to 0.13 ± 0.19 μM. There was no significant difference between both the BB3CMU-treated groups and the DF-treated group (*p* < 0.05).

It has been established that NO may govern different aspects of hair biology [8]. A mixture of several herbal extracts or the Yonnyuniksoogobon-dan formulation has been shown to stimulate hair growth and reduce inflammation via the downregulation of iNOS and transforming growth factor-beta on hair roots [46]. A subsequent study showed the hair-growth-promoting effects of *Laminaria japonica* and *Cistanche tubulosa* extract and an anti-inflammatory effect associated with the suppression of NO and PGE2 [47].

Phenolic compounds have been identified to possess anti-inflammatory activities due to their ability to scavenge free radicals and inhibit pro-inflammatory enzymes such as lipoxygenase, cyclooxygenase (COX), and NOS [40]. Similar findings were observed by Saji et al.: rice bran phenolic extracts, at a concentration of 0.025–0.25 mg/mL, lowered the level of NO, malondialdehyde, intracellular reactive oxygen species, and pro-inflammatory cytokine in RAW264.7 macrophage cells [30]. This study suggested that the synergistic action of phenolic compounds in rice bran extracts, including caffeic acid, ethyl vanillate, ferulic acid, feruloyl glycoside, *p*-coumaric acid, shikimic acid, sinapic acid, syringic acid, tricin, and vanillic acid, contributed to anti-inflammatory and antioxidant activities [30]. Additionally, flavonoids and flavonoid-rich extracts have been reported to suppress the expression of iNOS and NO levels [43,45,48]. Anti-inflammatory effects through NF-κB have been observed by flavonoids that can be found in rice fractions, including daidzein, genistein, naringenin, isorhamnetin, kaempferol, and quercetin [16,19,43]. As indicated in Table 2, BB3CMU-H and BB3CMU-RB contained high amounts of phenolic compounds, including caffeic acid, ferulic acid, *p*-coumaric acid, naringenin, kaempferol, and quercetin. These explained why both extracts possess anti-inflammation activity.

Beharka et al., reported that vitamin E could block COX activity in macrophages by decreasing the formation of peroxynitrite, a product of NO and superoxide [49]. Another study reported that α-tocopheryl acetate, a form of vitamin E, diminished the NO production in rat alveolar macrophages, suggesting a reduction in damage to lung tissue [40]. The possible mechanism of this may be due to the NO-scavenging ability of vitamin E [29,40,41]. Interestingly, δ-tocotrienol isolated from rice bran has been reported to possess anti-inflammatory activity towards LPS-activated inflammatory mediators, including NO, IL-1β, IL-6, TNF-α, and IFN-γ, via the disruption of MAPK and peroxisome proliferator-activated receptor (PPAR) signaling [50]. Additionally, among the vitamin E components, γ-tocopherol, δ-tocopherol, and γ-tocotrienol possess superior antioxidant and anti-inflammatory activities than those of α-tocopherol [31]. Although the content of phenolic compounds in BB3CMU-RB was lower than BB3CMU-H, tocopherols might contribute to the inhibition of NO production. Moreover, this might lead to the attenuation of COX and secretion of other mediators.

Our results verified that BB3CMU-H and BB3CMU-RB significantly attenuated the inflammation via the reduction in NO production, which was statistically comparable to DF. These could be the synergistic reaction of phenolic compounds and tocopherols that were found in BB3CMU extracts. Further studies are required to comprehend the role of bioactive compounds in BB3CMU-H and BB3CMU-RB on specific molecular pathways of the inflammatory process.

### 2.5. Effect of BB3CMU-H and BB3CMU-RB on Gene Expression of Steroid 5α-Reductase Isoenzymes

The initial evidence that AGA results from the androgen pathway came from the observation of men with testicular insufficiency [51]. The baldness and reduced sebum production in skins and hairs were observed in eunuchoid and prepubertally castrated men [51]. It is currently accepted that AGA is associated with androgen sensitivity and the increase in DHT accumulation in the genetically predisposed pilosebaceous units of individuals [2].

The intracellular conversion of testosterone into DHT occurs via 5α-reductase enzymes. Three isotypes of 5α-reductase enzymes are types 1, 2, and 3, which are encoded by the genes *SRD5A1*, *SRD5A2*, and *SRD5A3*, respectively [2,52]. Commonly prescribed medicines of AGA treatment are 5α-reductase inhibitors, including finasteride (a type 2 inhibitor) and dutasteride (a dual-type 1 and 2 inhibitor) [9]. Sawaya et al., found that the balding hair follicles expressed a higher activity of 5α-reductase enzymes type 1 and 2 than non-balding hair follicles [53]. Nevertheless, it was proposed that isotype 2 played a predominant role in AGA etiology [54]. In addition, AGA-prone tissues express high activity for 5α-reductase type 2, androgen receptor activation, and DHT [1]. Higher expression levels of 5α-reductase type 3 were found in skin and other peripheral tissues than types 1 and 2, advocating that it could regulate DHT production [55]. A recent study postulated that the expressions of *SRD5A1*, *SRD5A2*, and *SRD5A3* were higher in the anagen hairs plucked from female AGA than in the controls, suggesting the crucial role of *SRD5A* in AGA [52].

**Figure 2:**
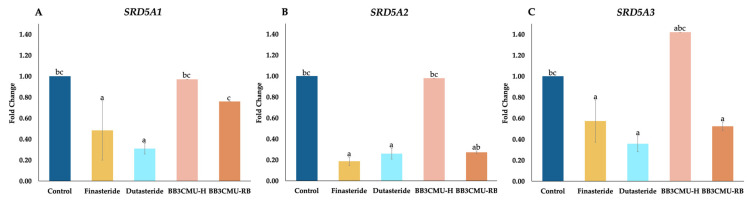
Effects finasteride, dutasteride, husk extract of *Oryza sativa* L. cv. Bue Bang 3 CMU (BB3CMU-H), and bran extract of *Oryza sativa* L. cv. Bue Bang 3 CMU (BB3CMU-RB) at the equal concentration of 0.10 mg/mL on mRNA expression of *SRD5A1* (**A**), *SRD5A2* (**B**), *SRD5A3* (**C**) in DU-145 cells for 24 h. Different letters indicate significant differences (*p* < 0.05): a, b, and c indicate statistical significance in comparison to control, finasteride, and dutasteride, respectively.

Many studies provided evidence that supports the hair-growth-promoting efficacy of herbal extracts and their bioactive compounds via the inhibition of 5α-reductase activity [9,56,57,58,59]. Only a few studied the downregulation of *SRD5A* expression, and reduction in the protein translation of 5α-reductase enzymes. DU-145 has been used as in vitro model to investigate the effects of substances on 5α-reductase activity and *SRD5A* expression [55,56,60,61,62]. Thus, this study evaluated the effect of BB3CMU-H and BB3CMU-RB on *SRD5A1*, *SRD5A2*, and *SRD5A3* expression, as shown in Figure 2.

It is noteworthy that BB3CMU-RB significantly downregulated *SRD5A2* (fold change 0.27 ± 0.01) and *SRD5A3* (fold change 0.52 ± 0.04) compared with the control group. In Figure 2B,C, no significant differences can be seen between BB3CMU-RB, finasteride, and dutasteride regarding the downregulation of *SRD5A2* and *SRD5A3*. However, *SRD5A1* was slightly suppressed by BB3CMU-RB, with a fold change of 0.76 ± 0.01 compared with the control group. Tocopherols and γ-oryzanol were identified to diminish the mRNA expressions of *SRD5A1*, *SRD5A2*, and *SRD5A3* [56]. Furthermore, it was established that linoleic acid, enriched in rice bran extract, was responsible for the downregulation of *SRD5A1* expression [60]. The positive linear correlation between linoleic acid and total unsaturated fatty acid contents, and the suppression of *SRD5A1* expression, was established [61].

The affinity of steroid substrate towards 5α-reductase type 2 was greater than that of type 1 isozyme, suggesting an anabolic role of type 2 in the androgen metabolism [63]. A molecular dynamics simulation of α-tocopherol showed the stable interaction inside a binding pocket of 5α-reductase type 2, similar to finasteride [56]. Additionally, unsaturated fatty acids (e.g., γ-linolenic acid, docosahexaenoic acid, α-linolenic acid, linoleic acid, palmitoleic acid, oleic acid, and myristoleic acid) were verified to inhibit the 5α-reductase activity, suggesting the regulation of the androgen pathway in target cells [64]. The bran fraction is well-known to contain high concentrations of fatty acids and edible lipids [25]; therefore, BB3CMU-RB might impede the effect of 5α-reductases.

Our study revealed that the mRNA expression levels of *SRD5A1*, *SRD5A2*, and *SRD5A3* were not significantly decreased by BB3CMU-H treatment. No earlier study looked at the effect of rice husk extracts on *SRD5A* gene expression. Nevertheless, a previous study reported that the mRNA levels of *SRD5A1*, *SRD5A2*, and *SRD5A3* were slightly decreased by the treatment of gallic acid [56]. There is controversy regarding whether phenolic compounds affect the downregulation of the *SRD5A1* gene [61]. Additionally, the study reported that the activity of 5α-reductase type 1 was attenuated by natural phenolic compounds, such as green tea EGCG, myricetin, quercetin, baicalein, and fisetin, whereas type 2 was favorably inhibited by other flavonoids, including biochanin A, daidzein, genistein, and kaempferol [65]. The bioactive compounds of BB3CMU-H were mainly phenolic and flavonoid components, which did not influence *SRD5A* gene expression, but might affect the 5α-reductase enzyme [56].

Despite androgens promoting hair growth in the beard and the axillary sites, they have an antagonistic effect that depresses hair production on the frontal and vertex scalp [2]. In addition, AGA-prone hair follicles are genetically predisposed to sensitize the presence of DHT, leading to hair follicle miniaturization, hair thinning, and ultimately AGA [1]. Interestingly, DHT involves sebum production and pro-inflammatory cytokine secretion, which are responsible for skin disorders such as acne vulgaris and AGA [1,2]. Patients suffering from AGA express a high level of sebum production, bacterial colonies, and porphyrin—a by-product derived from the lipid metabolism of *Propionibacterium acnes* (*P. acnes*)—in androgen-sensitive AGA tissues [1,66].

Perifollicular inflammation is triggered by oxidative stress, *P. acnes*, harbored in the pilosebaceous units, porphyrins, sebum, and androgenic stimuli [1,10]. These pathogenic factors may emphasize an androgen response feedback loop, AGA-mediated inflammation and oxidative stress, and pathogenic microorganisms. It is also interesting to note that DHT might enhance NF-κB-mediated TNF-α and TGF-β1 by increasing the production of ROS and NO, resulting in microinflammation [6]. Consequently, the attenuation of the androgen pathway could be a regulatory mechanism to modulate sebum production, bacterial colonies, inflammation, and oxidative stress.

BB3CMU-H was rich in phenolic compounds, polysaccharides, and proteins, with strong antioxidant capacities. On the other hand, tocopherols, γ-oryzanol, and fatty acids were the most abundant in BB3CMU-RB, resulting in better anti-androgenic activities regarding the downregulation of SRD5A genes and inhibition of 5α-reductase enzymes. Both BB3CMU-H and BB3CMU-RB-RB possessed anti-inflammation properties via the suppression of NO production. Therefore, the combination of BB3CMU-H and BB3CMU-RB could be applied for hair growth promotion or utilized as an adjuvant to the AGA treatment.

## 3. Materials and Methods

### 3.1. Chemicals and Reagents

Anthrone; 2,2′-azino-bis(3-ethylbenzothiazoline-6-sulfonic acid) diammonium salt (ABTS); 2,2-diphenyl-1-picrylhydrazyl (DPPH); bovine serum albumin; caffeic acid; catechin; chlorogenic acid; *o*-coumaric acid; *p*-coumaric acid; diclofenac sodium; epicatechin; (−)-epigallocatechin gallate (EGCG); ethylenediaminetetraacetic acid (EDTA); ferrozine; ferrous chloride; ferulic acid; Folin-Ciocalteu phenol reagent; gallic acid; *p*-hydroxybenzoic acid; kaempferol; lipopolysaccharide (LPS); naringenin; naringin; phytic acid; quercetin; rosmarinic acid; rutin; sulforhodamine B (SRB); trolox; α-, β-, γ-, and δ-tocopherols were from Sigma Chemical (St. Louis, MO, USA). Finasteride and dutasteride were from Wuhan W&Z Biotech (Wuhan, China). Acetic acid; dimethyl sulfoxide (DMSO); ethanol; formic acid; methanol; sulfuric acid; trichloroacetic acid; and other chemical substances were from RCI Labscan (Bangkok, Thailand). Agarose and 50X Tris/acetic acid/EDTA (TAE) were purchased from Bio-Rad Laboratories (Hercules, CA, USA). Dulbecco’s Modified Eagle Medium (DMEM; cat no. 31600083); fetal bovine serum (FBS; cat no. 16000044); and Roswell Park Memorial Institute medium (RPMI-1640; cat no. 31800022) were from Gibco Life Technologies (Thermo Fisher Scientific, Waltham, MA, USA). Penicillin/streptomycin solution (100X) was from Capricorn Scientific GmbH (Ebsdorfergrund, Germany). Other chemicals were analytical grade.

### 3.2. Plant Material and Extract Preparation

**Figure 3:**
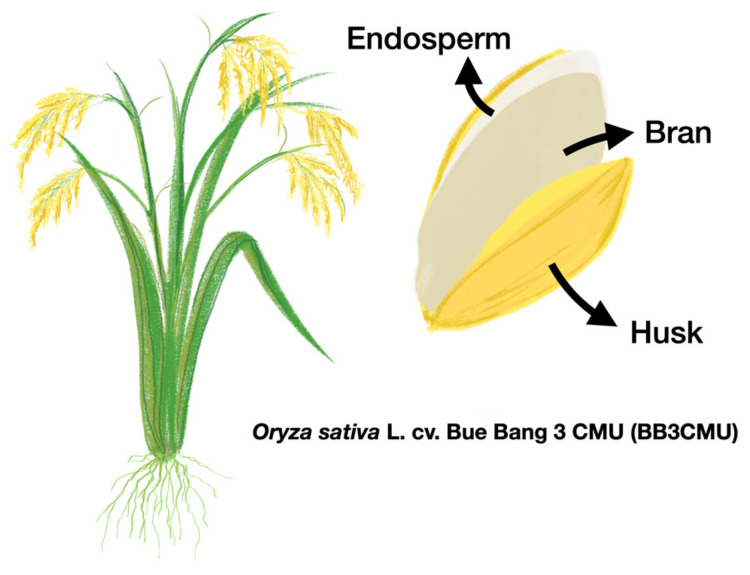
Composition of *Oryza sativa* L. cv. Bue Bang 3 CMU (BB3CMU).

The rice bran and husk fractions of *Oryza sativa* L. cv. Bue Bang 3 CMU (BB3CMU) used in the study were obtained from Lanna Rice Research Center, Chiang Mai University, Chiang Mai, Thailand, in December 2020 (Figure 3). The voucher specimens of rice bran (PNPRDU63025) and rice husk (PNPRDU63026) were deposited in the Pharmaceutical and Natural Products Research and Development Unit, Faculty of Pharmacy, Chiang Mai University. Two kilograms of each fraction were macerated in 95% (*v/v*) ethanol (1:3, *w/w*) for 24 h. The extract solutions were double-filtered through Whatman filter paper no. 4 and no. 1 and then vacuum-evaporated at 50 °C by an evaporator (Hei-VAP value, Heidolph, Schwabach, Germany). Ethanolic extracts of rice bran and husk were labeled as BB3CMU-RB and BB3CMU-H, respectively. Samples were stored at 4 °C until analysis.

### 3.3. Bioactive Compound Estimation

#### 3.3.1. Determination of Total Phenolic Content

Total phenolic content was determined according to the Folin–Ciocalteu method, with minor modifications [67]. The result was measured as milligrams of gallic acid equivalents per gram of extract (mg GAE/g extract).

#### 3.3.2. Determination of Total Flavonoid Content

The analysis of total flavonoid content was measured by the aluminum chloride colorimetric method, as described by Diniyaha et al. [68] with modifications. The result was determined as milligrams of EGCG equivalents per gram of extract (mg EGCGE/g extract).

#### 3.3.3. Determination of Total Polysaccharide Content

Total polysaccharide content was assayed as described by Luo et al. [69], with minor modifications using D-glucose as a standard. The result was determined in terms of milligrams of D-glucose equivalents per gram of extract (mg D-glucose/g extract).

#### 3.3.4. Determination of Total Protein Content

Total protein content of extracts was estimated by the Lowry method with some modifications [70]. BSA was used as a standard. Total protein content was reported in terms of milligrams of BSA equivalents per gram of extract (mg BSA/g).

### 3.4. Bioactive Characterization of Extracts

#### 3.4.1. Determination of Phenolic Compounds and Phytic Acid by Liquid Chromatography-Mass Spectrometry (LC-MS)

The separation and identification of phenolic compounds and phytic acid were performed using liquid chromatography (LC) (Agilent 1260 Infinity II series), equipped with an electrospray ion (ESI) quadrupole mass spectrometry 6130 (Agilent Tech., Santa Clara, CA, USA), according to the method of Mighri et al. [71], with minor modifications. Each sample (20 µL) was injected into a Restek Ultra C18 reversed-phase column (250 × 4.6 mm, 5 µm, Restek Corporation, Bellefonte, PA, USA). The mobile phase was A (0.2% acetic acid in 95% water and 5% methanol) and B (0.2% acetic acid in 50% water and 50% acetonitrile). The linear gradient elution was 10–20% B at 0–45 min, 20–55% B at 45–85 min, 55–100% B at 85–97 min, 100% B at 97–110 min, and re-equilibration of the column for 10 min using the initial conditions. The flow rate was 0.5 mL/min, and the column temperature was 40 °C. Spectra were recorded in the negative selected ion monitoring (SIM). Nitrogen gas was used as a nebulizer and an auxiliary gas. The rate of nebulizer and nitrogen flows was 1.5 L/min and 12 L/min, respectively. The ionization condition was operated at a capillary voltage of −3.5 V, a dissolving line temperature of 250 °C, a block source temperature of 400 °C, and fragmentation voltage of 70 V. The full scan MS spectra were in the range from 100 *m*/*z* to 1200 *m*/*z* with an acquisition rate of 250 ms/spectrum. Data acquisition and integration were operated using OpenLab software (Agilent Tech., Santa Clara, CA, USA).

#### 3.4.2. Determination of Tocopherols by High-Performance Liquid Chromatography (HPLC)

HPLC analysis was accomplished to quantify α-, β-, γ-, and δ-tocopherols using a Shimadzu HPLC system coupled with a fluorescence detector (RF-20A, Shimadzu Corporation, Kyoto, Japan), according to previous methods [72,73]. Chromatography separation of each sample was carried out using a normal-phase Inertsil SIL-100A packing column (5 μm, 4.6 × 250 mm, GL Sciences Inc., Tokyo, Japan). For tocopherols, an isocratic system was used. A linear gradient of the mobile phase consisting of 0.6% isopropanol in hexane was run for 30 min with a flow rate of 1 mL/min. The fluorescence acquisition wavelengths were set at 298 nm and 325 for excitation and emission, respectively. A comparison of the fluorescence signal obtained from samples and standards reference was carried out for tocopherol quantification.

### 3.5. Antioxidant Activities

#### 3.5.1. DPPH Radical Scavenging Assay

The DPPH radical scavenging capacity of each extract was carried out according to the previous method [74] with slight modifications. In brief, 150 μL of freshly prepared DPPH solution (0.1 mM) was mixed with 50 μL of different concentrations of trolox (0.02–0.4 mg/mL) or extract solution in a 96-well plate. After incubation for 30 min at room temperature in the dark, the absorbances were measured at 515 nm by a microplate reader (EZ Read 400 Flexi, Biochrom, Cambridge, UK). The measurement unit was expressed as milligrams of trolox equivalents per gram of extract (mg TE/g extract) using the trolox standard curve.

#### 3.5.2. ABTS Radical Cation Scavenging Assay

For the ABTS assay, the method was conducted based on the reported method [75]. The stock solution of ABTS^•+^ was prepared by mixing 7 mM of aqueous ABTS solution with 2.45 mM of aqueous potassium persulfate (2:1, *v/v*) and kept in the dark room for 16 h at room temperature. The mixture was diluted with ethanol until the absorbance at 734 nm reached 0.700 ± 0.020. Afterward, 160 μL of ABTS^•+^ working solution was reacted with varying concentrations of trolox (0.02–0.4 mg/mL) or extract solution, and absorbances at 734 nm were taken by a microplate reader (EZ Read 400 Flexi, Biochrom, Cambridge, UK) after incubation for 6 min. ABTS values were expressed in milligrams of trolox equivalents per gram of extract (mg TE/g extract).

#### 3.5.3. Metal Chelating Assay

Ferrous ion chelating activity was carried out using the described method [76] with some modifications. In brief, 50 μL of ferrous chloride (2 mM) was added to 100 μL of different concentration of EDTA (0.02–0.4 mg/mL) or extract solution in 96-well plate. The mixture was reacted with 50 μL of ferrozine (5 mM), and the absorbances at 515 nm were recorded by a microplate reader (EZ Read 400 Flexi, Biochrom, Cambridge, UK) after 10 min of incubation at room temperature. Results were expressed in terms of milligrams of EDTA equivalents per gram of extract (mg EDTAE/g extract).

### 3.6. Cell Culture Procedure

RAW 264.7 macrophage cells and DU-145 cells were obtained from American Type Culture Collection (Rockville, MD, USA). Cells were maintained in the incubator at 37 °C under 5% CO_2_. RAW 264.7 macrophage cells were grown in DMEM supplemented with 10% FBS and 1% penicillin/streptomycin. DU-145 cells were cultured in RPMI supplemented with 10% FBS and 1% penicillin/streptomycin. Cells between passages 20 and 30 were used in this study.

### 3.7. Cell Viability Assay

The non-cytotoxic concentration of extracts and cell viability of RAW 264.7 macrophage cells and DU-145 cells were determined using sulforhodamine B (SRB) colorimetric assay, as previously described [77]. Cells were seeded at 1 × 10^5^ cells/well in 96-well plates. After incubation for 24 h, cell monolayers were treated with various concentrations (0.2–2.5 mg/mL) of extracts, diclofenac sodium, finasteride, and dutasteride. After treatment for 24 h, cells were fixed with 50% (*w/v*) trichloroacetic acid and stained with 0.04% (*w/v*) SRB for 30 min. The unbound dye was washed using 1% (*v/v*) acetic acid. The protein-bound dye was solubilized with 10 mM of Tris base solution, and absorbances were read at 515 using a microplate reader. The absorbance of cells incubated in a treatment group was normalized to the absorbance of cells incubated in a control medium, which was considered 100% of cell viability. The highest non-toxic concentration with more than 80% cell viability was selected for further experiments.

### 3.8. Anti-Inflammatory Activity

Nitric oxide assay was used to evaluate the anti-inflammatory activity of extracts by measuring the nitrite, which is the final inert product of nitric oxide in the culture medium [41,78]. RAW 264.7 macrophage cells were seeded at a density of 1 × 10^5^ cells/well in 96-well plates and incubated for 24 h. The cells were then pretreated with extracts (0.1 mg/mL), diclofenac sodium (0.1 mg/mL), and solvent as a blank for 2 h. Subsequently, LPS (0 or 1 µg/mL) was administered to the cells to induce an inflammatory response. After incubation for 24 h, the nitric oxide production in the supernatant was measured by Griess reagent kit (Invitrogen, Thermo Fisher Scientific, Inc.) according to the manufacturer’s protocol. In brief, 20 μL of Griess reagent mixture and 150 μL of supernatant were mixed in a 96-well plate, incubated for 30 min at room temperature, and then the absorbance was measured at 570 nm.

### 3.9. Semi-Quantitative Reverse Transcription and Polymerase Chain Reaction

*SRD5A1*, *SRD5A2*, and *SRD5A3* are expressed in the DU-145 androgen-insensitive prostate adenocarcinoma cell line [55,79]. These cells have been used in previous studies to observe the regulation of SRD5A gene expression [56,61,80]. Total RNA was isolated from DU-145 cells treated with 0.1 mg/mL of the extracts (BB3CMU-H and BB3CMU-RB), 0.1 mg/mL of finasteride and dutasteride, or non-treated cells using the E.Z.N.A.^®^ Total RNA Kit I (Omega Bio-Tek, Georgia, USA), according to the manufacturer’s instructions. RNA quantity was measured by a Qubit™ 4 fluorometer (Invitrogen, Carlsbad, USA) and Qubit™ RNA HS Assay Kit (Invitrogen). The total RNA was stored at −20 °C until further analysis.

Gene expression levels were carried out by a semi-quantitative RT-PCR [81]. The extracted RNA samples were further subjected to RT-PCR Quick Master Mix (Toyobo, Osaka, Japan), according to the manufacturer’s instructions. The reaction contained 1 µg of total RNA, 10 µL of Master Mix, 1 µL of manganese acetate, and 1.2 µL of specific primers (Integrated DNA Technologies, Coralville, IA, USA).

Primer sequences used are as follows: *SRD5A1*: AGCCATTGTGCAGTGTATGC and AGCCTCCCCTTGGTATTTTG; *SRD5A2*: TGAATACCCTGATGGGTGG and CAAGCCACCTTGTGGAATC; *SRD5A3*: TCCTTCTTTGCCCAAACATC and TCCTTCTTTGCCCAAACATC; *GAPDH*: GGAAGGTGAAGGTCGGAGTC and CTCAGCCTTGACGGTGCCATG. Nucleic acid amplification was performed on DW-T960 Gradient PCR Thermal Cycler (Drawell, Shanghai, China) with the condition of a reverse transcription step for 30 min at 60 °C, pre-denaturation at 94 °C for 1 min, and 40 cycles of 30 s denaturation at 94 °C, 30 s annealing at 50–55 °C, and 1 min extension at 72 °C.

RT-PCR products were run on 1% agarose gel stained with RedSafe™ dye (iNtRON Biotechnology, Gyeonggido, Korea) in the chamber with 1X TAE running buffer. The quantification of bands was acquired by Gel Doc™ EZ System (Version 3.0; Bio-Rad, Hercules, CA, USA) and Image Lab™ software (Bio-Rad, Hercules, CA, USA). The expression of the target gene was normalized relative to *GAPDH*, compared with the control group, and expressed as the terms of the fold change.

### 3.10. Statistical Analysis

The results were presented as means of triplicate analysis. Statistical comparisons were carried out in Jamovi version 1.6.23 (The Jamovi Project, Sydney, Australia). The one-way analysis of variance (ANOVA), followed by a post-hoc analysis (Tukey’s test) and the independent samples *t*-test, were applied to compare means and evaluate statistical differences. The null hypothesis was rejected at the calculated probability value < 5%.

## 4. Conclusions

AGA is a chronic progressive condition caused by genetic predisposition and androgens, leading to hair follicle miniaturization. 5α-Reductase is the key enzyme that converts testosterone into DHT. In AGA-prone hair follicles, excessive androgen activities stimulate inflammation and oxidative stress. Consequently, a feedback loop is created and leads to chronic baldness. Conventional medicines have several drawbacks and a narrow mode of mechanisms. Herbal constituents have received attention due to their multiple modes of biochemical action, with fewer side effects. Rice husk and bran are generated during milling processes, which can cause serious environmental consequences. The novel utilization of agricultural wastes as a value-added product needed to be investigated. The non-glutinous local rice variety, Bue Bang 3 CMU (BB3CMU), which is grown in northern Thailand, was investigated in this study for its potential uses in cosmeceuticals, nutraceuticals, and pharmaceuticals. Our data revealed that BB3CMU husk extract (BB3CMU-H) possessed the most antioxidant activities due to the high amounts of phenolic compounds and phytic acid, whereas BB3CMU rice bran extract (BB3CMU-RB) was rich in α-, β-, γ-, and δ-tocopherol, giving it the ability to diminish the expression of *SRD5A* genes and attenuate 5α-reductase isozymes. Remarkably, both extracts attenuated NO productions in macrophages, which could help to slow down the inflammatory response and ROS production. However, further studies are required to fully decipher the effects of bioactive compounds in BB3CMU-H and BB3CMU-RB on specific molecular pathways. This study concluded that the synergistic effects of BB3CMU-H and BB3CMU-RB could be applied for the preventive or curative management of AGA, which supports the zero-waste principle, as they could be implemented as healthcare products and sustainable resources for pharmaceutical or cosmetical applications.

## Figures and Tables

**Table 1:** Bioactive compound estimation of BB3CMU-H and BB3CMU-RB.

Sample	Total Phenolic Content (mg GAE/g Extract)	Total Flavonoid Content (mg EGCGE/g Extract)	Total Polysaccharide Content (mg D-glucose/g Extract)	Total Protein Content (mg BSAE/g Extract)
BB3CMU-H	23.68 ± 0.56 ^a^	82.57 ± 9.89	0.22 ± 0.01 ^a^	0.40 ± 0.01 ^a^
BB3CMU-RB	11.63 ± 0.40 ^b^	81.64 ± 4.26	0.05 ± 0.01 ^b^	0.16 ± 0.01 ^b^

Note: husk extract of *Oryza sativa* L. cv. Bue Bang 3 CMU (BB3CMU-H); bran extract of *Oryza sativa* L. cv. Bue Bang 3 CMU (BB3CMU-RB); milligrams of gallic acid equivalents per gram of extract (mg GAE/g extract); milligrams of epigallocatechin gallate equivalents per gram of extract (mg EGCGE/g extract); milligrams of D-glucose equivalents per gram of extract (mg D-glucose/g extract); milligrams of bovine serum albumin equivalents per gram of extract (mg BSAE/g extract); ^a^ and ^b^ indicate significant differences (*p* < 0.05) in comparison to BB3CMU-RB and BB3CMU-H, respectively.

**Table 2:** Content of phenolic compounds, phytic acid, and tocopherols of BB3CMU-H and BB3CMU-RB.

Bioactive Compound	Molecular Formula	BB3CMU-H	BB3CMU-RB
		(μg/g Extract)	(μg/g Extract)
Caffeic acid	C_9_H_8_O_4_	105.5 ± 0.6	40.18 ± 0.01
Catechin	C_15_H_14_O_6_	295.4 ± 0.4	ND
Chlorogenic acid	C_16_H_18_O_9_	204.7 ± 3.7	113.8 ± 0.01
*o*-Coumaric acid	C_9_H_8_O_3_	910.1 ± 1.0	78.92 ± 0.01
*p*-Coumaric acid	C_9_H_8_O_3_	76.65 ± 3.90	99.80 ± 0.08
Epigallocatechin gallate	C_22_H_18_O_11_	163.6 ± 3.1	174.6 ± 0.01
Ferulic acid	C_10_H_10_O_4_	157.4 ± 5.4	22.87 ± 0.01
Gallic acid	C_7_H_6_O_5_	ND	16.84 ± 0.01
*p*-Hydroxybenzoic acid	C_7_H_6_O_3_	198.7 ± 2.8	ND
Kaempferol	C_15_H_10_O_6_	863.6 ± 2.1	34.23 ± 0.01
Naringin	C_27_H_32_O_14_	68.13 ± 1.74	113.2 ± 0.01
Phytic acid	C_6_H_18_O_24_P_6_	7358 ± 6.3	428.6 ± 0.01
Quercetin	C_15_H_10_O_7_	120.2 ± 0.3	34.27 ± 0.01
Rosmarinic acid	C_18_H_16_O_8_	51.43 ± 0.38	ND
Rutin	C_27_H_30_O_16_	150.9 ± 0.04	ND
α-tocopherol	C_29_H_50_O_2_	ND	854.3 ± 0.8
β-tocopherol	C_28_H_48_O_2_	ND	62.60 ± 0.20
γ-tocopherol	C_28_H_48_O_2_	ND	367.2 ± 1.3
δ-tocopherol	C_27_H_46_O_2_	ND	11.80 ± 0.40

Note: not detected (ND); husk extract of Oryza sativa L. cv. Bue Bang 3 CMU (BB3CMU-H); bran extract of *Oryza sativa* L. cv. Bue Bang 3 CMU (BB3CMU-RB).

**Table 3:** Antioxidant activities of BB3CMU-H and BB3CMU-RB.

Sample	DPPH Assay (mg TE/g Extract)	ABTS Assay (mg TE/g Extract)	Metal Chelation (mg EDTAE/g Extract)
BB3CMU-H	14.64 ± 1.30 ^a^	19.94 ± 0.65 ^a^	6.85 ± 4.53 ^a^
BB3CMU-RB	8.61 ± 0.01 ^b^	9.16 ± 0.19 ^b^	90.92 ± 4.11 ^b^

Note: husk extract of *Oryza sativa* L. cv. Bue Bang 3 CMU (BB3CMU-H); bran extract of *Oryza sativa* L. cv. Bue Bang 3 CMU (BB3CMU-RB); milligrams of trolox equivalents per gram of extract (mg TE/g extract); milligrams of ethylenediaminetetraacetic acid equivalents per gram of extract (mg EDTAE/g extract); ^a^ and ^b^ indicate significant differences (*p* < 0.05) in comparison to BB3CMU-RB and BB3CMU-H, respectively.

## Data Availability

The data presented in this study are available on request from the corresponding author.
